# Extensive Cutaneous and Scalp Metastases From Hormone Receptor-Positive Breast Cancer Successfully Treated With CDK4/6 Inhibitor Therapy: A Case Report

**DOI:** 10.7759/cureus.108234

**Published:** 2026-05-04

**Authors:** Ricardo Fernández-Ferreira, Verónica Bautista-Piña, Mireya Barragán-Dessavre, Georgina Cruz-Morales, Jesús Montaño-Hernández, Alejandro Salas-Pérez, Rosa María Vicuña-González, Rosa Anaya-Jiménez, Regina Moreno-López, Sandra-María García-López, Isabel-Alicia Loya-Aguilar

**Affiliations:** 1 Department of Medicine, Universidad Nacional Autónoma de México, Mexico City, MEX; 2 Department of Oncology Medicine, Hospital Central Sur de Alta Especialidad PEMEX, Mexico City, MEX; 3 Department of Oncology Medicine, Hospital Médica Sur, Mexico City, MEX; 4 Department of Pathology, Hospital Central Sur de Alta Especialidad PEMEX, Mexico City, MEX; 5 Department of Dermatology, Hospital Central Sur de Alta Especialidad PEMEX, Mexico City, MEX

**Keywords:** breast cancer metastasis, cdk4/6 inhibitor, cutaneous metastasis of breast cancer, cutaneous scalp metastasis, hormonal therapy

## Abstract

Cutaneous metastases from breast cancer (CMBC), including those involving the scalp, are typically observed in advanced stages of the disease and are relatively uncommon, particularly in hormone receptor-positive subtypes.

We present the case of a 68-year-old woman with breast cancer metastatic to the skin, characterized by multiple firm, hyperpigmented, painless nodules distributed over the body, including the scalp. The patient has been treated with a CDK4/6 inhibitor (ribociclib) in combination with endocrine therapy (ET) (letrozole) since April 2024.

During follow-up, all subcutaneous nodular lesions showed significant regression. By April 2026, skin color had returned to normal in most previously affected areas, and the majority of nodules had resolved. The scalp lesion completely disappeared, with full hair regrowth at the affected site.

Treatment of CMBC with CDK4/6 inhibitor-based therapy has been scarcely reported, with most cases describing ET alone. This case highlights the potential role of CDK4/6 inhibitors and documents a notable response in the scalp, skin, and subcutaneous tissue, which has not been widely described.

## Introduction

Female breast cancer (BC) is the second most common cancer worldwide, with an estimated 2.3 million new cases in 2022, accounting for 11.6% of all cancer diagnoses. It is also the fourth leading cause of cancer-related mortality, with approximately 666,000 deaths (6.9% of all cancer deaths) [1]. In Mexico, BC represents a major public health problem, with an incidence rate of 27.9% [1,2]. Approximately 30% of patients experience disease recurrence within five years; of these, 36% present with local recurrence, and 56% with metastatic disease [3].

BC metastases may occur in common clinical sites such as bone (30%-68.8%), lung and pleura (16%-32%), liver (13.3%-32%), and brain (1.9%-10%), but may also involve less common sites such as the scalp (approximately 2%) [4-8]. Cutaneous metastases occur in 0.7%-10.4% of patients with cancer [9,10]. In BC, cutaneous metastases are detected at the time of primary diagnosis in 6.3% of cases and represent the initial clinical presentation in 3.5% of patients [6,10].

Patients with hormone receptor-positive metastatic BC are generally diagnosed at a later stage and are commonly treated with endocrine therapy (ET). The use of aromatase inhibitors (anastrozole and letrozole) has been reported in this setting; however, there are limited reports describing treatment with modern CDK4/6 inhibitor-based therapy in this clinical presentation [3-10].

We present a case of metastatic BC with generalized cutaneous involvement, characterized by multiple firm, hyperpigmented, painless nodules in a patient with a history of breast surgery. The patient was treated with a CDK4/6 inhibitor-based regimen, achieving a favorable response and good tolerability.

This article was previously presented as an abstract at the 2025 San Antonio Breast Cancer Symposium (SABCS), held on December 12, 2025.

## Case presentation

A 68-year-old woman with a significant gynecologic history (two pregnancies and six months of oral contraceptive use) presented for evaluation.

Comorbidities

Chronic obstructive pulmonary disease secondary to smoking, treated with prednisone 5 mg daily, tiotropium bromide inhaler, inhaled salmeterol/fluticasone (50/100 μg), and nightly supplemental oxygen. Major depressive disorder, treated with paroxetine 20 mg daily and clonazepam 2 mg daily.

She had a history of right BC diagnosed in 2000, clinical stage IIB without high-risk features for recurrence, treated with right radical mastectomy followed by adjuvant hormonal therapy with letrozole 2.5 mg daily for five years.

In October 2023, she presented to the Emergency Department with dyspnea at rest, asthenia, adynamia, bone pain, and disseminated dermatosis. In November 2023, an excisional biopsy of a subcutaneous nodule was performed, revealing moderately differentiated invasive ductal carcinoma. Immunohistochemistry showed estrogen receptor positivity (95%), progesterone receptor positivity (90%), HER2 negativity, and Ki-67 expression in 30% of tumor cells (Figures 1A-1E).

**Figure 1:**
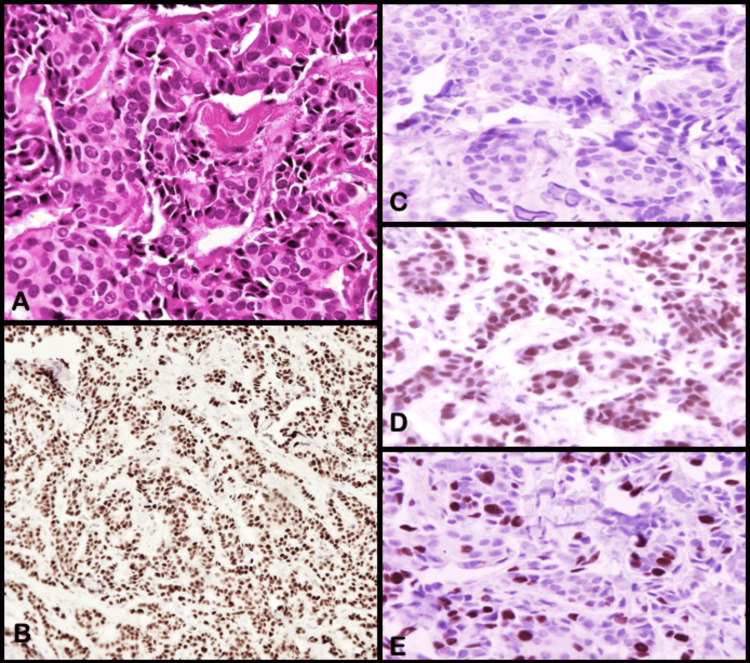
Microscopic examination in November 2023 showing metastatic ductal carcinoma involving the reticular dermis, with intense and diffuse expression of estrogen receptors (A-B) Immunohistochemistry revealed HER2-negative expression (C), intense nuclear expression of estrogen receptors and progesterone receptors (D), and Ki-67-positive expression in 30% of the neoplastic cells (E).

The patient was seen for medical oncology evaluation in April 2024 and was found to have an Eastern Cooperative Oncology Group (ECOG) performance status of 2 and a Karnofsky Performance Status of 70%.

Patient-reported outcomes (PROs) were assessed at treatment initiation using the EORTC QLQ-C30 and EORTC QLQ-BR23 questionnaires [11,12], which indicated poor quality of life (QoL). The G8 (Geriatric 8) score was 13, consistent with a frail patient.

Laboratory studies showed hemoglobin of 11.2 g/dL, platelet count of 208,000/μL, glucose of 120 mg/dL, and creatinine of 0.36 mg/dL. Elevated lactate dehydrogenase (LDH) levels and a rapid increase in bilirubin to >1.5× the upper limit of normal were observed in the absence of Gilbert’s syndrome or biliary tract obstruction.

An electrocardiogram showed sinus rhythm, a heart rate of 70 bpm, and a QTc of 422 ms. Given the presence of visceral disease (liver metastases, pleural effusion, and dyspnea), poor functional status, and the comparable efficacy of CDK4/6 inhibitors to chemotherapy in this setting, combination therapy with endocrine treatment was selected.

In April 2024, treatment was initiated with ribociclib 600 mg orally once daily and letrozole 2.5 mg orally once daily, in combination with denosumab. The regimen was well tolerated, with no reported toxicities.

During treatment, a partial response was observed on contrast-enhanced CT scans of the chest, abdomen, and pelvis. Figures 2A-2C show the subcutaneous nodular lesions in April 2024, compared with those in Figures 2D-2E from May 2025.

**Figure 2:**
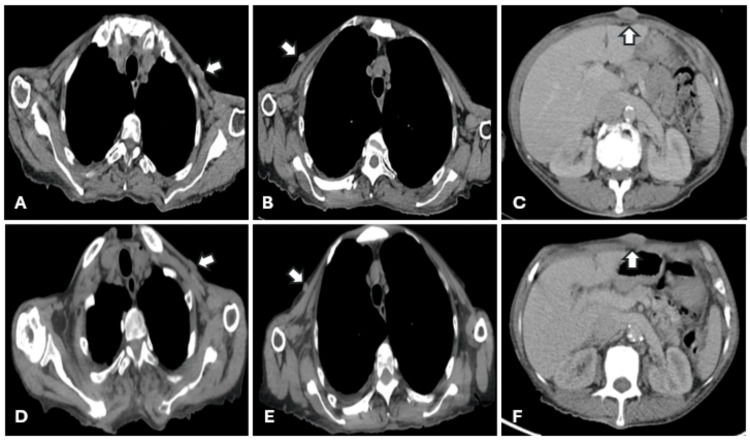
Chest and abdominal CT scans Chest and abdominal CT scans show subcutaneous lesions in the left supraclavicular region in April 2024 (A), with a decrease in size in May 2025 (D). In the right anterior thorax, another subcutaneous nodule is seen in April 2024 (B), with minimal reduction in size in October 2025 (E). In the abdominal region, a subcutaneous nodule is observed in April 2024 (C), showing a decrease in size and flattening in October 2025 (F).

A decrease in pulmonary metastases and pleural effusion was also observed on follow-up CT imaging (Figures 3A-3B vs. Figures 3D-3E), along with a reduction in hepatic metastases (Figure 3C vs. Figure 3F).

**Figure 3:**
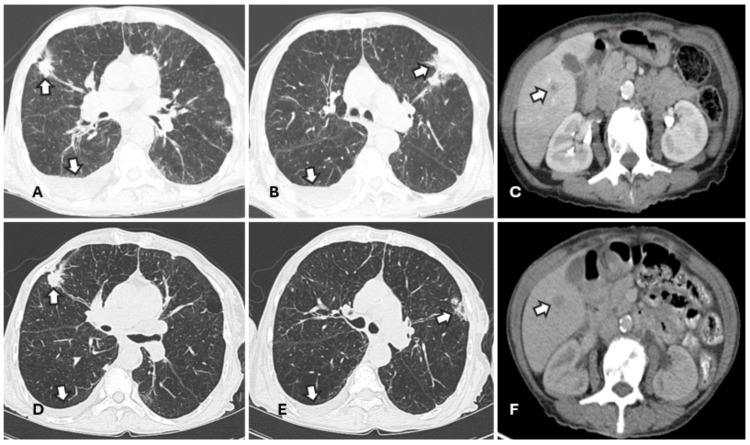
Chest CT scans Chest CT scans showing lung metastases and pleural effusion in April 2024 (A-B), with a significant decrease in October 2025 (D-E). Abdominal CT scans demonstrated liver metastasis in April 2024 (C), with a reduction in the lesion in October 2025 (F).

The scalp showed multiple subcutaneous nodular lesions associated with alopecia. The firm, stony, painless subcutaneous nodules measured approximately 1-3 cm and were located on the anterior and posterior chest, abdomen, and back (Figures 4A-4C).

**Figure 4:**
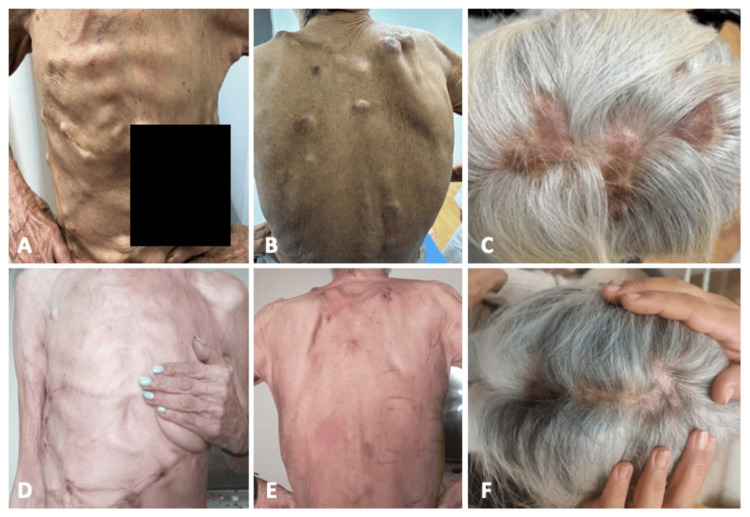
Patient images In April 2024, she presented with multiple 1-3 cm subcutaneous nodules on the anterior chest wall and abdominal region (A), which had significantly decreased in size by April 2026 (D). She also had 1-5 cm subcutaneous nodules in the left supraclavicular region and posterior chest, adjacent to the scapulae and spine (B), which had almost completely resolved by April 2026 (E). On the scalp, multiple brown subcutaneous nodules measuring approximately 3-5 cm were observed, associated with alopecia in these areas in April 2024 (C). By April 2026, the nodules had disappeared, and hair had regrown in the affected areas (F).

During follow-up, all subcutaneous nodular lesions showed significant regression. Within the first three to four months after treatment initiation, the lesions became flat, with residual brownish hyperpigmentation at the affected sites. As of April 2026, the skin color had returned to normal in most previously involved areas, and most nodules had resolved. The scalp lesion had completely disappeared, with complete hair regrowth at the affected site (Figures 4D-4F).

The most recent imaging study, a whole-body FDG PET/CT scan performed in February 2026, demonstrated a partial response, with a significant (>30%) reduction in lesions in the lungs, liver, and bones (Figures 5A-5D).

**Figure 5:**
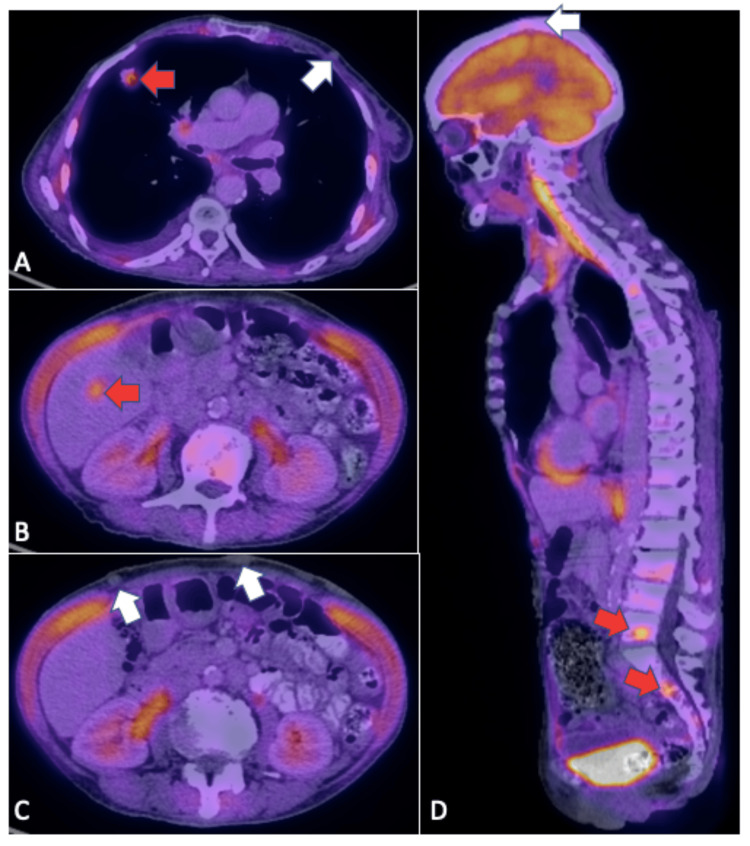
FDG PET/CT scan Red arrows indicate a partial response in the lung (A), liver (B, C), and bone metastases (D), as well as resolution of pleural effusion (A). White arrows indicate complete response in subcutaneous nodular lesions of the scalp, anterior and posterior thoracic regions, and abdomen (A-D). FDG-PET/CT: Fluorodeoxyglucose positron emission tomography/computed tomography

At her last medical evaluation in February 2026, the patient was receiving ribociclib 600 mg orally once daily for 21 days of a 28-day cycle in combination with letrozole 2.5 mg orally once daily, completing 21 months of treatment.

Her performance status was ECOG 1, with a Karnofsky Performance Status of 90%. PROs, assessed using the EORTC QLQ-C30 and EORTC QLQ-BR23 questionnaires, showed an improvement in QoL. The G8 score remained 13, consistent with a frail patient.

## Discussion

Approximately 6% of patients with BC present with de novo metastatic disease, and 14% develop recurrent metastatic disease [6-10,13]. Cutaneous metastases from breast cancer (CMBC) are relatively common, particularly in recurrent disease; however, their presence as part of widely disseminated disease is generally considered a sign of disease progression or advanced metastatic spread [14].

Notably, 69% of cutaneous metastases in women evaluated by dermatologists originate from BC and are present in approximately 24% of patients with metastatic BC [15]. The most common sites of skin metastasis include the anterior chest wall and abdomen. Other reported sites include the contralateral breast, surgical scars, upper extremities, the head and neck region, and the scalp, with rare involvement of the buttocks, perianal region, lower extremities, and eyelids. Scalp metastasis from BC is exceedingly rare, occurring in fewer than 2% of patients, while combined disseminated cutaneous and scalp involvement has been reported in approximately 7.8% of cases [15-18].

In general, metastatic recurrence involving the skin or subcutaneous tissue is often the initial finding that prompts medical evaluation, as it is frequently detected during self-examination. Currently, surveillance for early-stage BC after five years typically includes annual clinical and imaging follow-up. As a result, visceral metastatic recurrence may be detected late, leading to advanced-stage presentation, as observed in our patient.

The median age of women with BC presenting with cutaneous metastases is 74 years. Unfortunately, at the time of diagnosis, most cutaneous metastases are associated with widespread disease, and curative treatment is generally not feasible [6-18].

The diagnosis of CMBC is often delayed or missed because clinical manifestations are highly variable and may mimic benign conditions. Cutaneous metastases typically present as solitary or multiple firm, pink to erythematous nodules ranging from 1 to 3 cm.

The clinical spectrum of CMBC is broad, ranging from the most common papulonodular pattern to rarer presentations such as dermatitis-like metastases. Zosteriform-like metastatic lesions have also been described; however, they do not follow a dermatomal distribution and are typically characterised by papules and nodules rather than vesicular or pustular lesions [19,20].

Cutaneous metastases may present with a variety of clinical morphologies. The most typical presentation consists of painless, firm, or elastic nodules that develop over a short period of time. Other reported manifestations include plaques, papules, nodules, erythematous or pigmented lesions, and inflammatory skin changes [17-22].

Damaskos et al. reported that the most frequent clinical manifestations were papules and/or nodules (80%), followed by telangiectatic carcinoma (11.2%), erysipeloid carcinoma (3%), carcinoma en cuirasse (3%), neoplastic alopecia (2%), and a zosteriform pattern (0.8%) [23].

The clinical presentation of our patient consisted of multiple nodular lesions measuring 1-3 cm, involving the trunk (anterior and posterior thoracic regions as well as the abdomen and back), while sparing the upper and lower extremities.

In the telangiectatic pattern, aggregates of atypical neoplastic cells and erythrocytes are present within dilated vessels of the papillary and/or reticular dermis [10].

Erysipeloid carcinoma presents as erysipelas-like lesions, characterised by sharply demarcated erythematous patches and plaques involving the breast and surrounding skin. Its incidence ranges from 3% to 6.3% among affected patients [24]. Carcinoma en cuirasse, also known as scirrhous carcinoma, appears as multiple firm, erythematous, indurated plaques over the chest wall [25]. Neoplastic alopecia is characterised histologically by tumor cells arranged in cords, with single cells infiltrating and destroying hair follicles and inducing fibroplasia [26].

BC scalp metastases typically present as one or more inflammatory or nodular lesions, telangiectasias, or irregularly shaped pigmented lesions, which may mimic other cutaneous malignancies and therefore be difficult to recognise clinically. Scalp metastases may also ulcerate or become secondarily infected [8,17,18].

Metastatic recurrence to the skin or subcutaneous tissue is not uncommon; however, involvement of the scalp and widespread cutaneous dissemination is rare. In our patient, scalp subcutaneous nodular lesions were associated with alopecia and, during treatment, became flat with brownish pigmentation, without evidence of ulceration or infection. After nine months of treatment, the lesions resolved and the scalp returned to normal appearance.

The strengths of this study include the fact that treatment response in the skin and subcutaneous tissue is easily visible and objectively measurable, leaving little doubt regarding therapeutic efficacy in these areas.

Another strength is that this report provides relevant dermatological information on the evolution of cutaneous and subcutaneous lesions during treatment from a morphological perspective, including changes in colour and the presence or absence of hair regrowth.

The limitations of this study include the fact that phase III clinical trials evaluating treatments in the metastatic setting, including patients with skin involvement, have not reported dedicated subgroup analyses to specifically assess the benefit of these therapies in this population. Another limitation is the scarcity of recent evidence (including descriptive studies, meta-analyses, cohort studies, and randomised trials) specifically addressing this clinical presentation, as CDK4/6 inhibitors have primarily been studied in the context of visceral metastatic BC over the past decade [17,22,25].

The diagnosis of cutaneous and scalp metastases can be challenging and requires a comprehensive clinical evaluation, along with non-invasive imaging techniques such as whole-body FDG PET/CT when available, or CT imaging in selected cases [27,28]. However, the gold standard for diagnosis remains histopathological and immunohistochemical analysis [29].

Kong et al. reported that at the time of cutaneous metastasis diagnosis, 56.8% of patients had more than one visceral metastatic site, most commonly involving bone (41.6%), lung (36%), liver (13.6%), and brain (1.6%) [30]. Therefore, a complete staging workup is essential for the timely detection of visceral metastases.

In our patient, CT demonstrated multiple visceral metastases involving the liver and lungs, as well as bone and subcutaneous lesions. During follow-up, an FDG PET/CT scan performed in February 2026 showed a significant reduction in the previously reported lesions on CT imaging.

Pathology

CMBC involves the dermis and/or subcutaneous tissue [19]. Histologically, these lesions are composed of atypical neoplastic cells arranged in small nests and cords, often infiltrating in a single-file pattern within the collagen bundles of the dermis, accompanied by a variable degree of fibrosis [9]. Immunohistochemical positivity for CK7, estrogen receptor, progesterone receptor, mammaglobin, TRPS1, and GATA3 supports the diagnosis of BC metastasis [31-33].

CMBC and histological-molecular subtypes of BC

In the series reported by Mayer et al., 95.2% of cutaneous metastasis cases originated from invasive carcinoma of no special type, followed by invasive lobular carcinoma (2.4%) and other histological subtypes (2.4%) [34].

It has been reported that the most common molecular subtype of CMBC is hormone receptor-positive/HER2-negative disease (47%-61%), similar to the case presented here, while less frequent subtypes include triple-negative (26%) and HER2-positive disease (10%) [30,35,36].

Treatment

In many cases of cutaneous metastases, the primary malignancy has already progressed to an advanced stage and is therefore not amenable to surgical treatment. Local therapies are primarily palliative and focus on symptom control, including wound care and hemostasis. Surgical excision may be performed in selected cases. In addition, local ablative treatments such as radiotherapy and electrochemotherapy are commonly used in the palliative setting to reduce symptoms associated with cutaneous metastases [25,37].

Currently, adjuvant treatment with CDK4/6 inhibitors in hormone receptor-positive and HER2-negative early BC is recommended only for patients at high risk of recurrence, including those with anatomical stage IIA disease with nodal involvement (N+), or node-negative disease (N0) with additional high-risk features, such as grade 2 or higher disease and a Ki-67 proliferation index of at least 20%, or high-risk genomic scores (Oncotype DX recurrence score ≥26, or high-risk classification by Prosigna PAM50, MammaPrint, or EndoPredict). It is also recommended for patients with stage IIB and stage III disease, according to the AJCC 8th edition criteria [38,39].

The use of aromatase inhibitors (anastrozole and letrozole) in CMBC has been reported in 13 case reports and appears to be particularly effective in estrogen receptor-positive disease [38,39].

In hormone receptor-positive metastatic BC, sequential lines of endocrine-based therapy are recommended until the development of endocrine resistance, including in cases of rapid disease progression or visceral crisis, defined as severe organ dysfunction [40].

To delay or overcome endocrine resistance, CDK4/6 inhibitors (palbociclib, ribociclib, and abemaciclib) and mechanistic target of rapamycin (mTOR) inhibitors (everolimus) have been evaluated and approved in this setting [41].

Everolimus improves progression-free survival (PFS) by approximately five months but has not demonstrated an overall survival (OS) benefit. In contrast, CDK4/6 inhibitors significantly improve PFS by approximately 10 months in the first-line setting and approximately five months in the second-line setting [42].

First-line treatment with palbociclib in combination with the nonsteroidal aromatase inhibitor (NSAI) letrozole did not demonstrate a statistically significant improvement in OS compared with letrozole alone in postmenopausal patients in the PALOMA-2 trial. Median OS was 53.9 vs 51.2 months (hazard ratio (HR) 0.96; 95% CI 0.78-1.18; p = 0.34) [43].

At eight years of follow-up, the MONARCH 3 trial showed that patients receiving abemaciclib plus an aromatase inhibitor achieved a median OS of 66.8 months compared with 53.7 months in the control arm in the intention-to-treat population, corresponding to an absolute difference of 13.1 months; however, this difference did not reach statistical significance (HR 0.804; 95% CI 0.637-1.015; p = 0.0664) [44].

In patients with visceral metastases, median OS exceeded five years in the abemaciclib arm, with an increase of 14.9 months compared with the control arm (63.7 vs 48.8 months). This subgroup included patients with liver and lung metastases; however, the OS difference was also not statistically significant (HR 0.758; 95% CI 0.558-1.030; p = 0.0757) [44].

In the MONALEESA-2 trial, ribociclib plus letrozole demonstrated a significant OS benefit compared with placebo plus letrozole. Median OS was 63.9 months versus 51.4 months (HR for death, 0.76; 95% CI, 0.63-0.93; two-sided p = 0.008). Similarly, in MONALEESA-7, conducted in premenopausal or perimenopausal patients, median OS was 58 months versus 48 months (HR 0.76; 95% CI 0.61-0.96) [45].

In MONALEESA-3, involving postmenopausal women with hormone receptor-positive/HER2-negative advanced BC receiving first-line ribociclib plus fulvestrant, the longest median OS reported was 67.6 months versus 51.8 months with placebo plus fulvestrant (HR 0.67; 95% CI 0.50-0.90) [46].

First-line ribociclib plus ET demonstrated a significant PFS benefit, similar response rates, and better tolerability compared with combination chemotherapy (docetaxel plus capecitabine, paclitaxel plus gemcitabine, or capecitabine plus vinorelbine) in patients with clinically aggressive hormone receptor-positive/HER2-negative advanced BC (HR+/HER2- ABC).

Median PFS was 21.8 months (ribociclib plus ET; 95% CI 17.4-26.7) versus 12.8 months (combination chemotherapy; 95% CI 10.1-18.4), with a hazard ratio of 0.61 (95% CI 0.43-0.87; p = 0.003). Overall response rates and median time to response were 66.1% versus 61.8%, and 4.9 months versus 3.2 months, respectively (HR 0.76; 95% CI 0.55-1.06) [47].

Treatment with modern CDK4/6 inhibitor-based therapy has not been specifically reported in CMBC, particularly in phase III trials or dedicated subgroup analyses. Our case demonstrates that this therapeutic approach may provide effective disease control not only in visceral and bone metastases but also in extensive cutaneous and subcutaneous involvement, achieving a marked clinical response.

If cutaneous metastases do not respond to ET, chemotherapy may be considered in these subtypes. However, no specific chemotherapy regimen for CMBC has been established, and the available evidence is limited to case reports and case series [48,49].

Prognosis

CMBC is associated with a poor prognosis, particularly in the presence of concomitant visceral metastases. They are often considered a preterminal event [16,49]. Schoenlaub et al. reported a median survival of 13.8 months after the diagnosis of CMBC; however, survival exceeding 10 years was observed in two cases [50-52].

## Conclusions

Our case suggests that treatment with CDK4/6 inhibitors may achieve a meaningful response in extensive hormone receptor-positive BC with scalp involvement. Metastatic involvement of the scalp is often associated with alopecia in the affected area; however, hair regrowth may occur in responders to treatment. CDK4/6 inhibitors in combination with ET represent a feasible and clinically beneficial treatment option for patients with cutaneous metastases from hormone receptor-positive BC, with an overall manageable safety profile, although evidence is primarily derived from limited clinical data.
